# Mitral Regurgitation after TAVR: Sex Differences and Low-Flow Low-Gradient Aortic Stenosis

**DOI:** 10.1016/j.jacadv.2026.103140

**Published:** 2026-09-23

**Authors:** Eric D. Warner, Serge Harb, Rhonda Miyasaka, Rishi Puri, Amar Krishnaswamy, Samir R. Kapadia, Grant W. Reed

**Affiliations:** Heart, Vascular and Thoracic Institute, Cleveland Clinic, Cleveland, Ohio, USA

**Keywords:** low-flow low-gradient aortic stenosis, mitral regurgitation, sex differences, transcatheter aortic valve replacement, valvular heart disease

## Abstract

**Background:**

Moderate or severe mitral regurgitation (MR) is common in patients with severe aortic stenosis (AS) treated with transcatheter aortic valve replacement (TAVR).

**Objectives:**

The objectives of the study was to help define the prevalence and prognosis of persistent MR post-TAVR, particularly in women and in low-flow low-gradient (LFLG) AS.

**Methods:**

In this single-center, observational study, 344 consecutive TAVR patients with severe AS and moderate or greater MR pre-TAVR were identified. Patients were stratified by AS subtype (classic vs LFLG). Factors associated with MR improvement to mild or less were evaluated using multivariable logistic regression. Survival was analyzed with Cox proportional hazards models adjusted for clinical and echocardiographic covariates.

**Results:**

The mean age was 81.9 years, 48.5% were females, and 45.1% had LFLG AS. MR improved by ≥1 grade in 62.8% and to mild or less in 52.9% of patients, with similar rates in classic vs LFLG AS. On multivariable analysis, severe (vs moderate) baseline MR, hypertension, and female sex were independently associated with lower odds of MR improvement to mild or less. In LFLG AS, female sex and severe MR remained significant predictors of persistent MR, whereas a higher pre-TAVR aortic valve mean gradient was associated with improved MR. Residual severe MR was associated with increased mortality, driven by those with LFLG AS.

**Conclusions:**

More than half of TAVR patients with moderate or severe MR experience MR improvement post-TAVR. Women and those with severe MR are less likely to have a significant improvement in MR. Patients with LFLG AS and persistent moderate or severe MR have worse survival post-TAVR.

Severe aortic stenosis (AS) is frequently associated with concomitant moderate to severe mitral regurgitation (MR), with some studies indicating a rate as high as 33% in patients undergoing transcatheter aortic valve replacement (TAVR).^1^ In the PARTNER A and B trials, the rate of significant MR was 19.8% and 22.2% respectively.^2^^,^^3^ Although it has been well established that the risk of mortality for patients undergoing TAVR with significant MR is higher than that of those with single valve disease, there are little data to define the management of patients with these dual valve lesions.^4^, ^5^, ^6^ As TAVR is now approved at all levels of surgical risk, it is important to identify optimal treatment strategies for these patients.^7^

Although surgical aortic valve replacement (SAVR) allows simultaneous treatment of valvular lesions, treatment of polyvalvular disease is associated with higher perioperative morbidity and mortality, making a heart team approach critical.^8^^,^^9^ Options can range from surgical AVR and mitral valve repair/replacement, TAVR with medical management of MR, or TAVR with plans for a transcatheter mitral valve intervention at a later date vs simultaneous intervention. These decisions are made even more complex in patients with low-flow low-gradient (LFLG) AS, when it can be difficult to discern if the patient’s symptoms are primarily due to AS vs MR, and how to sequentially address these lesions.

Although previous studies have investigated periprocedural changes in MR and the effect on mortality, most have had limited ability to predict MR improvement to mild or less.^10^^,^^11^ The impact of mixed AS/MR has also been investigated in patients with LFLG AS, although these studies were small or had only short-term follow-up.^12^^,^^13^ To our knowledge, sex disparities in patients with concurrent AS/MR have never been investigated. This is critical to understand, as women tend to have higher rates of MR, LFLG AS, and worse outcomes after SAVR or TAVR.^12^^,^^14^, ^15^, ^16^ Accordingly, this study aims to evaluate the relationship between concomitant AS/MR, specifically focusing on classical vs LFLG AS status, and differences in MR improvement to mild or less between male and female patients.

## Methods

This study is a single-center, retrospective, observational analysis from the Cleveland Clinic TAVR database. This study was approved by the Cleveland Clinic Institutional Review Board; informed consent was waived given the retrospective nature of the analysis. From January 2016 to December 2020, 2,630 patients underwent TAVR. The decision to perform TAVR was made by a heart team based on established criteria. Only patients with severe AS based on an aortic valve area (AVA) <1 cm^2^ were included in this study. Patients who underwent valve-in-valve TAVR or TAVR for an indication other than AS, and those with aborted procedures or missing key data, were excluded. Patients with moderate or greater MR on pre-TAVR echocardiography were included.

Patients underwent transthoracic echocardiographic examinations before TAVR, at hospital discharge, at 1 to 2 months after TAVR, and at 1 year after TAVR. Aortic valve area was calculated in accordance with American Society of Echocardiography guidelines, primarily using the continuity equation. All echocardiograms were performed by certified sonographers and interpreted by board-certified echocardiographers. Follow-up time was defined as the time from the procedure to the last documented contact with the patient, if alive, or to the time of death. Clinical variables, demographics, and mortality were determined from a retrospective chart review of the electronic medical records system (Epic software; Epic Systems). MR severity was graded based on integrative assessment per American Society of Echocardiography guidelines and entered into the clinical database by the interpreting echocardiographer. The primary endpoint was improvement of MR to mild or less, assessed at the first postprocedural echocardiogram (at hospital discharge or the 1-2 month follow-up visit). Serial echocardiograms at 1 year were used to characterize the longitudinal durability of MR improvement.

Statistical analysis was performed with IBM SPSS Statistics version 29.0 (IBM Corp). Categorical baseline and procedural characteristics were presented as frequencies (percentage of patients), and continuous variables were presented as mean ± SD with comparisons across groups using chi-square tests or t-tests. To identify independent predictors of MR improvement to mild or less, a univariate screen was performed using binary logistic regression. Multicollinearity was assessed using the variance inflation factor; variables with variance inflation factor >5 were excluded from the final multivariable model. No retained variable exceeded this threshold. Variables with *P* values <0.25 were candidates to be included in the multivariate analysis, along with age and sex. Variable selection was based on established biological plausibility and consistent precedent in the TAVR and valvular disease literature; all included covariates have been independently associated with MR outcomes or post-TAVR prognosis in prior published work. Interaction terms were tested between ejection fraction (EF), MR, mean gradient, and left atrial volumetric index, given their known physiologic inter-relationships in the AS/MR population, to evaluate whether the relationship between these continuous variables was modified by other covariables. Univariate and multivariate analysis was then repeated in the subgroup of patients with LFLG AS defined by AVA of <1 cm^2^, mean gradient of <40 mmHg, and stroke volume index <35 mL/m^2^. The primary time-to-event outcome for survival analysis was all-cause mortality at 3 years. Survival analysis was performed using multivariate Cox proportional hazard modeling among the entire cohort, those with LFLG AS, and those with classic AS. Multivariable Cox proportional hazard modeling was adjusted for clinically selected variables, including age, female sex, coronary artery disease, Society of Thoracic Surgeons (STS) risk score, EF, postprocedural mean gradient, moderate or greater Tricuspid Regurgitation (TR) postprocedurally, and residual MR severity. The proportional hazards assumption was assessed using Schoenfeld residuals; no significant violations were identified in any model (all *P* > 0.09). Interaction terms between EF, MR severity, mean gradient, and left atrial volume index were assessed as multiplicative product terms and evaluated via likelihood ratio testing. Kaplan-Meier curves were performed comparing residual MR grades after TAVR, and pairwise comparisons were performed using log-rank testing. Patients who did not undergo a postprocedural echo were excluded from this portion of the analysis.

## Results

### Baseline clinical and echocardiographic characteristics

A total of 344 patients who underwent TAVR had moderate to severe MR at baseline. The average age was 81.9 years, and 167 (48.5%) were females. Hypertension (91%), atrial fibrillation/flutter (58.4%), coronary artery disease (56.7%), and chronic lung disease (43.9%) were the most common comorbidities. LFLG patients had higher STS risk scores (8.42 vs 7.23; *P* = 0.039) with no significant differences in other comorbidities. A total of 189 (54.9%) had classic AS (normal flow/normal gradient), whereas 155 (45.1%) had LFLG AS. A total of 265 (77%) had moderate MR and 79 (23%) had severe MR, with no significant difference in distribution of MR across classic and LFLG AS groups (*P* = 0.354). Patients with LFLG AS had slightly larger AVA (0.72 vs 0.66 cm^2^; *P* = 0.005) but lower mean gradients (28.9 vs 45.0 mm Hg; *P* < 0.001), lower EF (44.9% vs 53.7%; *P* < 0.001), and lower stroke volume index (24.6 vs 34.1 mL/m^2^; *P* < 0.001) (Table 1).

**Table 1:** Baseline Demographics, Comorbidities, and Echocardiographic Characteristics

	Total (N = 344)	Classic as (n = 189)	LFLG as (n = 155)	*P* Value
Age, y (mean ± SD)	81.91 ± 8.56	81.72 ± 8.44	82.14 ± 8.73	0.651
STS risk score (mean ± SD)	7.76 ± 5.22	7.23 ± 4.79	8.42 ± 5.64	0.039∗
BMI, kg/m^2^ (mean ± SD)	27.36 ± 5.56	27.55 ± 5.49	27.13 ± 5.66	0.494
eGFR (mL/min/1.73 m^2^, mean ± SD)	56.60 ± 21.36	56.35 ± 20.66	56.89 ± 22.23	0.819
Female, n (%)	167 (48.5%)	88 (46.6%)	79 (51.0%)	0.416
Hypertension, n (%)	313 (91.0%)	170 (89.9%)	143 (92.3%)	0.456
Diabetes, n (%)	127 (36.9%)	64 (33.9%)	63 (40.6%)	0.195
Atrial fibrillation/flutter, n (%)	201 (58.4%)	109 (57.7%)	92 (59.4%)	0.753
Prior stroke, n (%)	48 (14.0%)	25 (13.2%)	23 (14.8%)	0.668
Coronary artery disease, n (%)	195 (56.7%)	100 (52.9%)	95 (61.3%)	0.119
Chronic lung disease, n (%)	151 (43.9%)	83 (43.9%)	68 (43.9%)	0.993
Aortic valve area, cm^2^ (mean ± SD)	0.69 ± 0.17	0.66 ± 0.20	0.72 ± 0.14	0.005∗
Peak aortic velocity, m/s (mean ± SD)	3.96 ± 0.68	4.35 ± 0.62	3.51 ± 0.43	<0.001∗∗
Aortic mean gradient, mm Hg (mean ± SD)	37.78 ± 14.12	45.03 ± 14.31	28.94 ± 7.15	<0.001∗∗
Left ventricular ejection fraction, % (mean ± SD)	49.72 ± 13.89	53.65 ± 11.11	44.94 ± 15.41	<0.001∗∗
Stroke volume index, mL/m^2^ (mean ± SD)	29.76 ± 9.36	34.00 ± 9.54	24.59 ± 5.95	<0.001∗∗
Left atrial volume index, mL/m^2^ (mean ± SD)	55.51 ± 18.57	54.84 ± 18.78	56.27 ± 18.35	0.499
LV end-diastolic diameter, cm (mean ± SD)	4.85 ± 0.85	4.79 ± 0.85	4.93 ± 0.86	0.141
LVOT VTI, m (mean ± SD)	0.19 ± 0.05	0.21 ± 0.05	0.17 ± 0.05	<0.001∗∗
Dimensionless index, by VTI (mean ± SD)	0.21 ± 0.05	0.20 ± 0.05	0.22 ± 0.05	<0.001∗∗
Severe mitral regurgitation, n (%)	79 (23.0%)	47 (24.9%)	32 (20.6%)	0.354

BMI = body mass index; eGFR = estimated glomerular filtration rate; LFLG = low-flow low-gradient; LV = left ventricular; LVOT = left ventricular outflow tract; STS = Society of Thoracic Surgeons; VTI = velocity time integral.

∗ *P* < 0.05,

∗∗ *P* < 0.001.

### Factors associated with improvement of MR

MR improved by at least 1 grade after TAVR in 216 patients (62.8%). Rates of improvement of MR were similar between classic and LFLG AS (63.0% vs 62.6%; *P* = 0.942), in patients with moderate or severe MR (59.5% vs 63.8%; *P* = 0.490), and patients with normal vs reduced EF (65.7% vs 58.7%; *P* = 0.190). Patients with low stroke volume index demonstrated slightly greater MR improvement compared with those with normal stroke volume index (65.9% vs 53.5%; *P* = 0.039).

**Figure 1:**
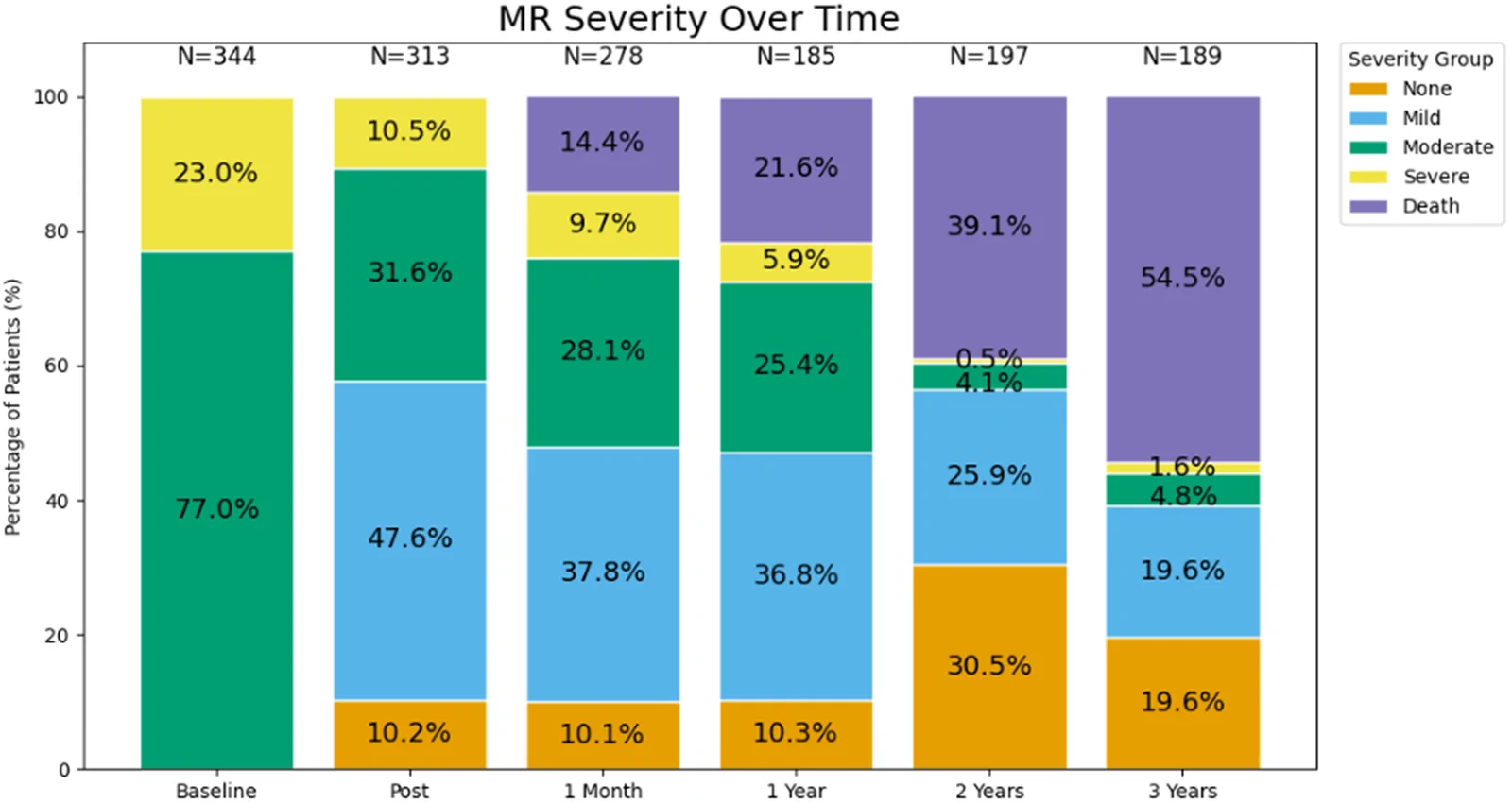
Severity of Mitral Regurgitation Over Time Bar chart displaying changes in the severity of mitral regurgitation (MR) over time. The majority of patients who survived to 3 years had mild or less MR while over half did not survive (purple). Death reflects patient vital status at the time of follow-up and is not a category of MR severity.

MR improved to mild or better after TAVR in 182 patients (52.9%), with similar rates in both LFLG and classic AS (53.4% vs 52.9%; *P* = 0.827) and between males and females (53.8% vs 46.2%; *P* = 0.347). MR severity progressively improved following TAVR, with sustained reductions observed over follow-up (Figure 1).

**Figure 2:**
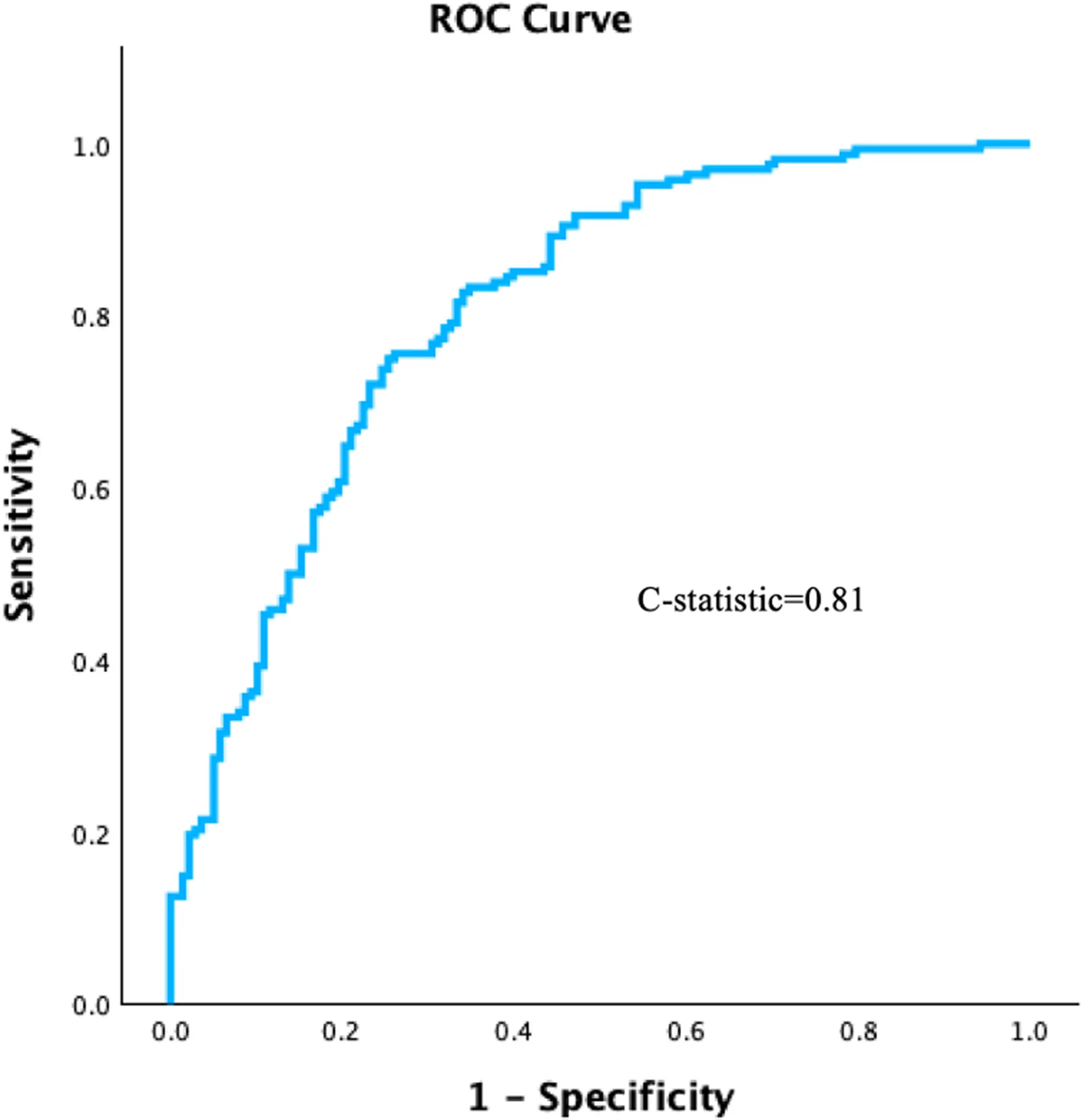
Discriminative Performance of Patients Achieving Mild or Less Mitral Regurgitation C-statistic curve demonstrating discriminative performance of the multivariable logistic regression model for patients who achieved MR improvement to mild or less. The C-statistic was 0.81 (95% CI: 0.78-0.87, bootstrap 1,000 resamples), reflecting good model discrimination. The curve was constructed using predicted probabilities from the full multivariable model presented in Table 2. This model has not been externally validated and should be considered exploratory. ROC = receiver operating characteristic.

On the univariate screen among all patients, several clinical and echocardiographic variables were associated with MR improvement to mild or less (Supplemental Table 1). On multivariate analysis, pre-TAVR severe MR was strongly associated with decreased odds of MR improvement to mild or less (OR: 0.06; 95% CI: 0.02-0.14; *P* < 0.001). Female sex (OR: 0.49; 95% CI: 0.27-0.90; *P* = 0.022) and hypertension (OR: 0.26; 95% CI: 0.08-0.80; *P* = 0.019) were also associated with lower odds of MR improvement to mild or less (Table 2). None of the prespecified interaction terms reached statistical significance, there was no interaction between EF and baseline MR severity (*P* = 0.429), EF and aortic valve mean gradient (*P* = 0.763), aortic valve mean gradient and MR grade at baseline (*P* = 0.965), or left atrial volumetric index and MR grade (*P* = 0.778). Receiver operating characteristic curve analysis demonstrated good discriminative performance of the multivariable model for identifying patients who achieved MR improvement to mild or less, with a C-statistic of 0.81 (95% CI: 0.78-0.87, bootstrap 1,000 resamples) (Figure 2).

**Table 2:** Multivariate Analysis of Factors Associated With MR Improvement to Mild or Less Among All Patients (Classic + LFLG Combined)

	OR (95% CI)	*P* Value
Age (per year)	1.02 (0.98-1.06)	0.345
Female	0.49 (0.27-0.90)	0.022∗
Prior pacemaker/ICD	0.91 (0.45-1.84)	0.798
Hypertension	0.26 (0.08-0.80)	0.019∗
Atrial fibrillation/flutter	0.95 (0.50-1.80)	0.868
STS risk score (per point)	0.95 (0.88-1.02)	0.182
eGFR (per mL/min/1.73 m^2^)	1.00 (0.99-1.02)	0.612
Aortic valve area (per cm^2^)	0.48 (0.04-5.72)	0.561
Chronic lung disease	0.72 (0.40-1.30)	0.279
Aortic valve mean gradient (per mm Hg)	1.02 (0.99-1.05)	0.282
Left ventricular ejection fraction (per %)	1.00 (0.97-1.03)	0.922
Left atrial volume index (per mL/m^2^)	1.00 (0.98-1.02)	0.869
LV end-diastolic diameter (per cm)	0.87 (0.59-1.28)	0.481
LVOT VTI (per cm)	1.03 (0.95-1.11)	0.515
Severe MR (vs moderate MR)	0.06 (0.02-0.14)	<0.001∗∗
≥ Moderate tricuspid regurgitation	0.76 (0.42-1.39)	0.374

ICD = implantable cardioverter-defibrillator; MR = mitral regurgitation; other abbreviations as in Table 1.

∗ *P* < 0.05,

∗∗ *P* < 0.001.

On univariate screen among patients with LFLG AS, several clinical and echocardiographic variables were again associated with MR improvement to mild or less (Supplemental Table 2). Among patients with LFLG AS, on multivariate analysis, female sex (OR: 0.36; 95% CI: 0.16-0.83; *P* = 0.016) and severe MR (OR: 0.14; 95% CI: 0.05-0.44; <0.001) remained independently associated with reduced odds of MR improvement to mild or less. A higher aortic valve mean gradient was independently associated with MR improvement (OR: 1.08 per mmHg; 95% CI: 1.02-1.15; *P* = 0.011) (Table 3). The C-statistic of 0.81 (95% CI: 0.78-0.87) reflects the discriminative performance of the overall model; the curve in Figure 2 was constructed using predicted probabilities from the full multivariable logistic regression model in Table 2.

**Table 3:** Multivariable Model of Factors Associated With MR Improvement to Mild or Less Among Patients With LFLG AS

	OR (95% CI)	*P* Value
Age (per y)	1.04 (0.99-1.09)	0.118
Female	0.36 (0.16-0.83)	0.016∗
Coronary artery disease	0.51 (0.23-1.13)	0.096
Aortic valve mean gradient (per mm Hg)	1.08 (1.02-1.15)	0.011∗
≥ Moderate tricuspid regurgitation	0.50 (0.23-1.12)	0.092
Severe MR (vs moderate MR)	0.14 (0.05-0.44)	<0.001∗∗
STS risk score (per point)	0.95 (0.87-1.03)	0.186
Left ventricular ejection fraction (per %)	1.01 (0.98-1.04)	0.655

AS = aortic stenosis; other abbreviations as in Tables 1 and 2.

∗ *P* < 0.05,

∗∗ *P* < 0.001.

### Residual MR and survival after TAVR

**Figure 3:**
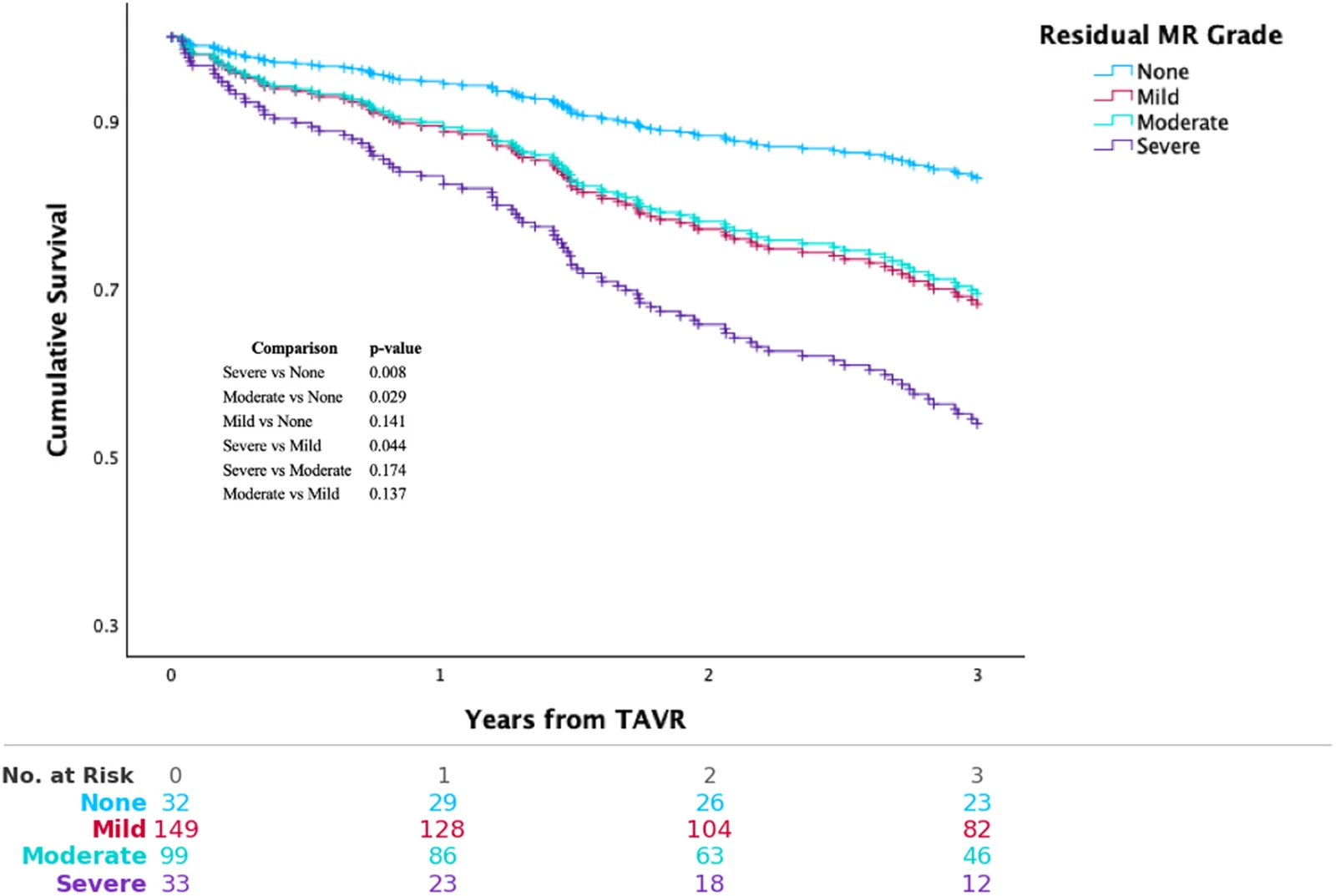
Survival by Residual Mitral Regurgitation Grade Kaplan-Meier survival curves with pairwise comparisons for residual mitral regurgitation grade for all patients (classic and low-flow low-gradient aortic stenosis combined). Residual severe and moderate MR was associated with increased mortality vs no MR. MR = mitral regurgitation; TAVR = transcatheter aortic valve replacement.

In multivariable Cox proportional hazards analysis, when evaluating all patients, in whom there were 103 deaths, (classic and LFLG together), residual severe MR (vs none) was associated with significantly higher mortality (HR: 3.35; 95% CI: 1.07-10.47; *P* = 0.037), whereas mild (HR: 2.08; 95% CI: 0.74-5.85; *P* = 0.166) and moderate residual MR (HR: 1.98; 95% CI: 0.68-5.79; *P* = 0.211) were not. STS risk score was also associated with increased mortality (HR: 1.06 per point; 95% CI: 1.03-1.10; *P* < 0.001), but age, EF, coronary artery disease, post-TAVR mean gradient, and female sex were not (Table 4). Kaplan-Meier curves with pairwise comparisons were similar, but showed both severe (*P* = 0.008) and moderate (*P* = 0.029) residual MR was associated with increased mortality relative to no MR (Figure 3).

**Table 4:** Cox Regression Predicting Post-TAVR Survival Among All Patients

	HR (95% CI)	*P* Value
Age	1.01 (0.98-1.03)	0.669
Female	0.77 (0.48-1.24)	0.280
Coronary artery disease	0.83 (0.53-1.31)	0.421
STS risk score (per point)	1.06 (1.03-1.10)	<0.001∗∗
Left ventricular ejection fraction (per %)	0.99 (0.97-1.01)	0.241
Mean gradient at discharge (per mm Hg)	0.98 (0.93-1.03)	0.407
≥ Moderate TR post	1.08 (0.68-1.70)	0.749
Residual MR severity		
Mild MR (vs none)	2.08 (0.74-5.85)	0.166
Moderate MR (vs none)	1.98 (0.68-5.79)	0.211
Severe MR (vs none)	3.35 (1.07-10.47)	0.037∗

TR = tricuspid regurgitation; other abbreviations as in Tables 1 and 2.

∗ *P* < 0.05,

∗∗ *P* < 0.001.

**Figure 4:**
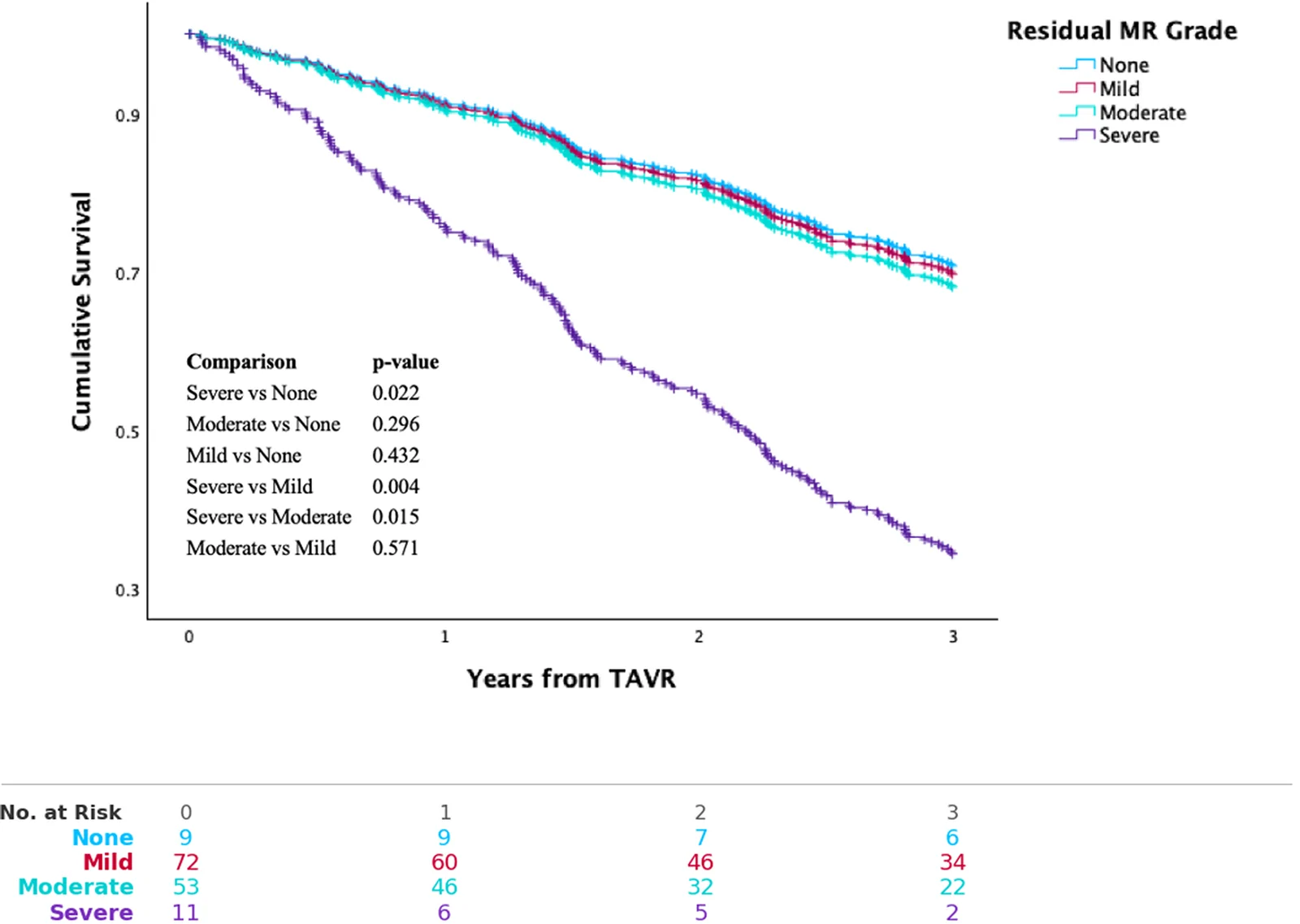
Survival by Residual Mitral Regurgitation Grade in Low-Flow Low-Gradient Aortic Stenosis Kaplan-Meier survival curves with pairwise comparisons for residual mitral regurgitation grade for patients with low-flow low-gradient aortic stenosis. Residual severe MR was associated with increased mortality vs moderate, mild, and no MR. Abbreviations as in Figure 3.

**Figure 5:**
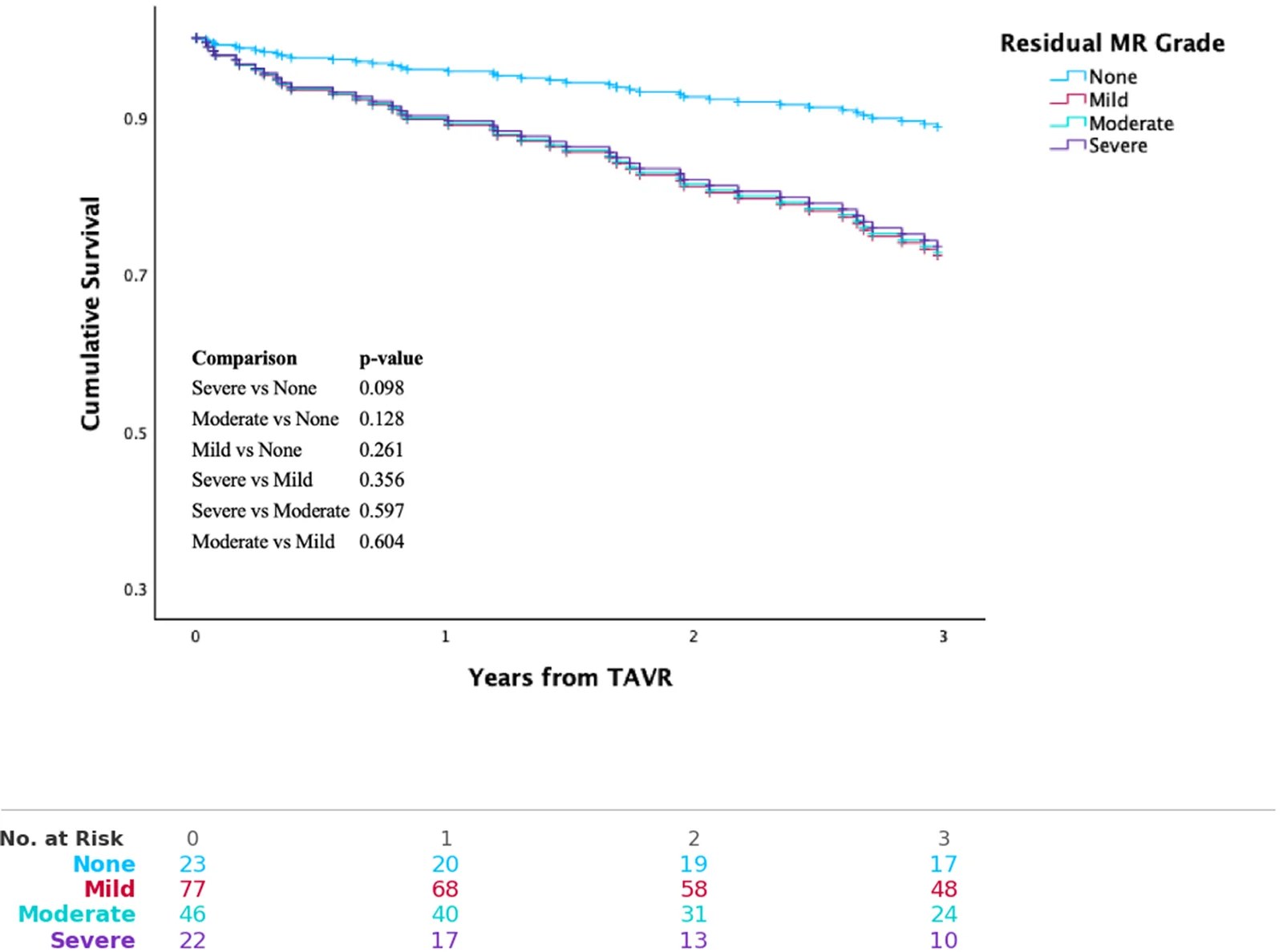
Survival by Residual Mitral Regurgitation Grade in Classic Aortic Stenosis Kaplan-Meier survival curves with pairwise comparisons for residual mitral regurgitation (MR) grade for patients with classic aortic stenosis. There is a trend toward worsening survival with severe MR; however, this was not statistically significant. Abbreviation as in Figure 3.

Significant differences emerged when evaluating the association of MR with mortality in classical and LFLG AS patients. Among patients with LFLG AS, among whom there were 56 deaths, Cox proportional hazards modeling showed residual severe MR (vs none) was associated with significantly higher mortality (HR: 3.08; 95% CI: 1.39-6.83; *P* = 0.006), whereas mild (HR: 1.04; 95% CI: 0.75-1.45; *P* = 0.805) and moderate residual MR (HR: 1.11; 95% CI: 0.67-1.83; *P* = 0.683) were not. Age, EF, coronary artery disease, post-TAVR mean gradient, moderate or greater tricuspid regurgitation, and female sex were not significantly associated with mortality (Table 5). Kaplan-Meier curves with pairwise comparisons showed increased mortality with severe vs moderate (*P* = 0.015), mild (*P* = 0.004), and no (*P* = 0.022) MR (Figure 4). In contrast, among patients with classic AS, among whom there were 47 deaths, residual MR severity was not associated with increased mortality on Cox regression or on Kaplan-Meier analysis (Table 6, Figure 5).

**Table 5:** Cox Regression Predicting Survival After TAVR Among LFLG Patients

	HR (95% CI)	*P* Value
Age	1.01 (0.99-1.03)	0.503
Female	0.84 (0.60-1.17)	0.298
Coronary artery disease	0.78 (0.57-1.07)	0.125
Left ventricular ejection fraction (per %)	0.99 (0.98-1.00)	0.152
Mean gradient at discharge (per mm Hg)	0.98 (0.94-1.01)	0.229
≥ Moderate TR post	1.14 (0.78-1.64)	0.503
Residual MR severity		
Mild MR (vs none)	1.04 (0.75-1.45)	0.805
Moderate MR (vs none)	1.11 (0.67-1.83)	0.683
Severe MR (vs none)	3.08 (1.39-6.83)	0.006∗
STS risk score (per point)	1.07 (1.04-1.10)	<0.001∗∗

Abbreviations as in Tables 1 and 2.

∗ *P* < 0.05,

∗∗ *P* < 0.001.

**Table 6:** Cox Regression Predicting Survival After TAVR Among Classic AS Patients

	HR (95% CI)	*P* Value
Age	0.97 (0.93-1.01)	0.091
Female	0.57 (0.27-1.19)	0.136
Coronary artery disease	0.76 (0.38-1.52)	0.434
Left ventricular ejection fraction (per %)	0.99 (0.96-1.02)	0.447
Mean gradient at discharge (per mm Hg)	1.05 (0.96-1.13)	0.285
≥ Moderate TR post	1.07 (0.53-2.16)	0.855
Residual MR severity		
Mild MR (vs none)	2.70 (0.62-11.83)	0.188
Moderate MR (vs none)	2.66 (0.58-12.23)	0.210
Severe MR (vs none)	2.57 (0.50-13.22)	0.260
STS risk score (per point)	1.12 (1.05-1.20)	<0.001∗∗∗

∗*P* < 0.05, ∗∗*P* < 0.001.

Abbreviations as in Tables 1 and 2.

**Central Illustration:**
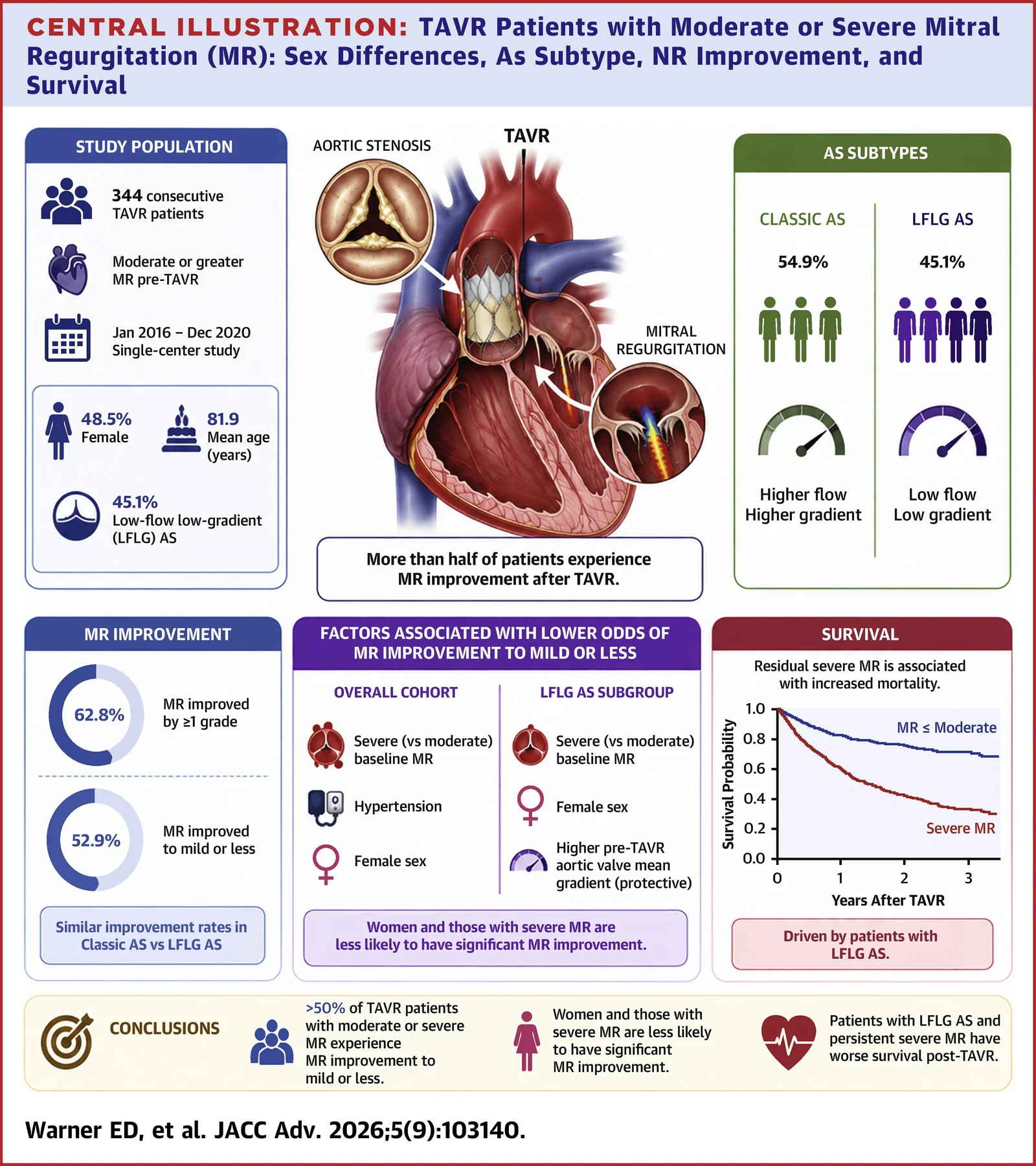
TAVR Patients with Moderate or Severe Mitral Regurgitation (MR): Sex Differences, As Subtype, NR Improvement, and Survival Among 344 patients with severe aortic stenosis and moderate or severe mitral regurgitation (MR) undergoing transcatheter aortic valve replacement (TAVR), MR improved by at least 1 grade in 62.8% and to mild or less in 52.9% of patients. Severe baseline MR, female sex, and hypertension were associated with lower odds of MR improvement. In patients with low-flow low-gradient (LFLG) aortic stenosis, higher pre-TAVR mean gradient predicted MR improvement. Residual severe MR was associated with increased mortality, primarily among patients with LFLG aortic stenosis. AS = aortic stenosis; AVA = aortic valve area; LFLG = low-flow low-gradient; MR = mitral regurgitation; TAVR = transcatheter aortic valve replacement.

## Discussion

To our knowledge, this is the first study to examine differences in predictors of residual MR among patients with classical and LFLG severe AS treated with TAVR, with longitudinal follow-up out to 3 years. We found that almost 63% of patients improved MR by at least 1 grade, and 53% improved to mild or less MR with no difference between classic or LFLG AS phenotypes. This decrease in MR is consistent with the prior literature, with some studies reporting as low as 28% improvement and others as high as 80% with most in the 40% to 50% range.^1^^,^^4^^,^^13^^,^^17^^,^^18^ Importantly, the improvement in MR was sustained, with most patients surviving to 3 years having mild or less MR. Our results suggest that an immediate post-TAVR reduction in MR is often durable to intermediate-term follow-up.

Although a few studies have examined the impact of residual MR on survival in TAVR patients, none have specifically evaluated those with LFLG AS.^10^^,^^19^ Our results suggest that the impact of residual MR is different in patients with classical and LFLG severe AS treated with TAVR. Specifically, although there was a trend toward worse 3-year mortality with mild or worse MR in patients with classic AS (Figure 5), this did not reach significance. In contrast, in patients with LFLG, mild or moderate residual MR was not associated with higher mortality, although survival was considerably worse in patients with severe MR (Figure 4). A plausible pathophysiologic relationship for this finding is that patients with LFLG severe AS typically have reduced left ventricular (LV) function, LV dilation, and may be more tolerant to higher loading conditions due to chronic valvular heart disease. This also has important implications, as it suggests that severe MR should be the threshold for intervention for residual MR post-TAVR given the association with poor survival at 3-years. It is also important to note that LFLG status did not predict improvement of MR after TAVR.

Although a multitude of studies have attempted to predict improvement in MR after TAVR, results have been inconsistent, and predictors have ranged from complex, nonroutine computed tomography–derived mitral valve geometry to echocardiographic features and MR etiology, with some studies not identifying any predictors at all.^19^, ^20^, ^21^, ^22^, ^23^ Most of these studies have been small, and to our knowledge, none have shown a relationship between sex and post-TAVR MR outcomes. This is crucial as sex disparities in AS are quite prevalent. Women tend to have significant delays in diagnosis, are more frequently in a critical preoperative state, and have higher rates of concomitant MR.^12^^,^^16^ Women are also more likely to present with LFLG AS and are less frequently referred to specialists or receive AVR.^14^^,^^20^^,^^21^ Among patients undergoing TAVR, women have also been shown to have longer waiting times, which was associated with increased mortality.^22^ Among patients with AS undergoing SAVR, women have also been consistently shown to have increased mortality.^15^^,^^23^ In our multivariable model, we found that aside from preprocedural MR severity and hypertension, female sex was the only factor independently associated with MR severity postprocedurally. Women were half as likely as men to have MR improve to mild or less, despite controlling for LV dimensions and atrial volumes, both of which have been associated with persistent MR in prior studies. Understanding that women are at higher risk of persistent MR should influence preprocedural counseling and could influence patient selection. It is important to acknowledge that persistent severe MR after TAVR may reflect more advanced underlying myocardial or atrial disease, rather than serve as an independent driver of mortality. Accordingly, the association between residual severe MR and worse survival in LFLG AS patients should be interpreted cautiously. Any decision regarding subsequent mitral valve intervention, whether transcatheter edge-to-edge repair or surgical correction, should be individualized based on MR mechanism, symptom burden, LV size and function, pulmonary pressures, mitral anatomy, frailty, and overall procedural candidacy.

### Study limitations

The primary limitation of this study was its retrospective, observational nature. It is possible that patients who were least likely to have MR improve were instead sent to surgery for definitive repair. In addition, given the limitations of the database, we were unable to compare primary vs functional MR, as etiology was not routinely collected. In addition, our analysis did not assess the mechanism of MR (degenerative vs functional), or the subtype of functional MR (ventricular or atrial). Although we were unable to directly compare, ventricular and atrial size were included, which may serve as surrogates for atrial/ventricular functional MR measures. We were also unable to assess anatomic considerations, such as posterior leaflet restriction, mitral annular calcification, the presence of a cleft or complex mitral valve anatomy, or the mechanism of MR improvement or medication changes made. MR severity was graded at the discretion of board-certified echocardiographers and was not subject to independent blinded review, which may introduce inter-rater variability, particularly in the complex hemodynamic setting of AS/MR. MR mechanism and etiology (primary/degenerative vs secondary/functional, and ventricular vs atrial subtypes of functional MR) were not systematically captured in our institutional database and therefore could not be analyzed. Granular echocardiographic data, including pulmonary pressures, right ventricular size and function, mitral annular calcification, coexistent mitral stenosis, and detailed mitral valve anatomy, were not available in our database, and their absence limits additional mechanistic insight and predictive value. We did not systematically ascertain which patients underwent transcatheter or surgical mitral valve intervention during the follow-up period. Patients who underwent mitral intervention would have had postprocedural MR severity altered independently of TAVR physiology, and their inclusion in serial MR analyses represents a potential limitation. In addition, longitudinal blood pressure data and antihypertensive medication changes were not systematically available; inadequate blood pressure control may represent a modifiable contributor to persistent MR after TAVR and is an important area for future investigation. The large OR for severe vs moderate baseline MR (OR: 0.06) likely reflects in part a structural threshold effect, as patients with severe baseline MR must achieve a multigrade reduction to meet the outcome definition of mild or less; this estimate should be interpreted with caution, given the observational design and potential residual confounding by MR mechanism. The multivariable logistic regression model in the LFLG AS subgroup included 8 covariates among 143 patients with 77 events, yielding an events-per-variable ratio of 9.6, marginally below the conventional minimum threshold of 10; estimates for covariates with wider confidence intervals in this subgroup model should be interpreted with appropriate caution, and replication in a larger LFLG AS cohort is warranted. Interaction analyses were exploratory; the study was not powered for interaction testing, and null interaction findings should be interpreted cautiously.

## Conclusions

In this study of patients with moderate-to-severe MR and concomitant severe AS undergoing TAVR, slightly over half had improvement in MR to mild or less. This improvement tended to be sustained out to 3 years. On multivariate analysis, the severity of baseline MR and female sex emerged as factors independently associated with persistent MR in both classic severe AS and LFLG severe AS. Residual severe MR was associated with significantly increased mortality among patients with LFLG AS but not among those with classic AS (Central Illustration).

Perspectives **COMPETENCY IN MEDICAL KNOWLEDGE:** In patients with severe aortic stenosis and concomitant moderate or severe mitral regurgitation (MR) undergoing TAVR, MR improves to mild or less in about half of patients, and persistent MR may reflect the combined contribution of baseline MR severity, aortic stenosis hemodynamic phenotype, and sex. Recognizing that women and patients with severe baseline MR are less likely to experience MR regression, and that residual severe MR carries prognostic weight chiefly in low-flow low-gradient aortic stenosis, may help identify patients unlikely to improve with aortic valve intervention alone.

**TRANSLATIONAL OUTLOOK:** Prospective studies are needed to determine whether incorporating sex, aortic stenosis phenotype, and MR mechanism into pre-TAVR decision-making improves patient selection, refines the timing and role of staged mitral intervention, and enhances symptom relief and survival after aortic valve replacement.

## Funding support and author disclosures

Dr Harb served as a consultant/speaker for 10.13039/100001316Abbott Laboratories, 10.13039/100006520Edwards, 10.13039/100008497Boston Scientific, and TeraRecon. Dr Reed has received honoraria for consulting for 10.13039/100001316Abbott Laboratories, 10.13039/100008497Boston Scientific, and 10.13039/100006520Edwards Lifesciences. All other authors have reported that they have no relationships relevant to the contents of this paper to disclose.
