# Supplementary material for: Mitral Regurgitation after TAVR: Sex Differences and Low-Flow Low-Gradient Aortic Stenosis
Source: JACC Adv. 2026 Sep 23;5(9):103140. doi: 10.1016/j.jacadv.2026.103140 (PMC13616514; doi:10.1016/j.jacadv.2026.103140)
Supplement: Supplementary file 1 [file mmc1.pdf]

Supplementary Appendix:

Table S1. Univariate factors associated with MR improvement to mild or less among all patients

Variable	OR	95% CI	p-value
Age	1.001	0.977–1.026	0.925
Female	0.816	0.534–1.247	0.347
Prior PPM/ICD	0.726	0.435–1.213	0.222
CAD	0.782	0.509–1.200	0.260
Hypertension	0.504	0.230–1.106	0.087
Diabetes	0.852	0.550–1.321	0.475
History of Afib/Aflutter	0.668	0.434–1.030	0.068
STS Risk Score	0.900	0.856–0.946	<0.001**
BMI	1.009	0.971–1.048	0.663
eGFR (MDRD)	1.015	1.004–1.025	0.006**
Aortic Valve Area	0.306	0.088–1.061	0.062
Prior Stroke	0.962	0.523–1.772	0.902
Chronic Lung Disease	0.596	0.388–0.915	0.018*
Aortic Valve Peak Velocity	1.781	1.271–2.494	<0.001**
Aortic Valve Mean Gradient	1.032	1.015–1.049	<0.001**
LVEF	1.021	1.005–1.037	0.010*
Stroke Volume Index	0.987	0.965–1.010	0.262
LA Volume Index	0.986	0.974–0.999	0.029*
LVOT VTI	121.233	1.914–7678.422	0.023*
Dimensionless Index	0.007	0.000–0.459	0.020*
LV End-Diastolic Diameter	0.835	0.645–1.081	0.172
Baseline MR Severity	0.100	0.052–0.194	<0.001**
TR 2+ or more	0.585	0.378–0.903	0.016*
LFLG Status	0.954	0.623-1.459	0.827

Table S2. Univariate factors associated with MR improvement to mild or less among LFLG patients

Variable	Odds ratio	95% CI	p-value
Age (per year)	1.03	(0.99–1.07)	0.127
Female sex	0.64	(0.34–1.21)	0.169
Prior pacemaker/ICD	0.72	(0.35–1.48)	0.377
Coronary artery disease	0.48	(0.25–0.93)	0.029*
Hypertension	0.77	(0.23–2.53)	0.662
Diabetes	1.01	(0.53–1.92)	0.980
Atrial fibrillation/flutter	0.64	(0.34–1.23)	0.183
STS risk score (per point)	0.90	(0.84–0.97)	0.004
Body mass index (per kg/m²)	0.99	(0.94–1.05)	0.761
eGFR (per mL/min/1.73m²)	1.01	(1.00–1.02)	0.216
Aortic valve area (per cm²)	0.04	(0.004–0.46)	0.010
Prior stroke	1.00	(0.41–2.42)	0.993
Chronic lung disease	0.29	(0.15–0.56)	<0.001**
Aortic valve peak velocity	4.76	(2.04–11.11)	<0.001**
Aortic valve mean gradient (per mmHg)	1.10	(1.05–1.15)	<0.001**
Left ventricular ejection fraction (per %)	1.02	(1.00–1.04)	0.096
Stroke volume index (per mL/m²)	1.02	(0.96–1.07)	0.559
Left atrial volume index (per mL/m²)	0.99	(0.97–1.00)	0.119
LVOT VTI (per cm)	1.06	(0.98–1.14)	0.132
Dimensionless index	0.92	(0.86–0.99)	0.025*
LV end-diastolic diameter (per cm)	0.77	(0.53–1.13)	0.188
Severe MR (vs Moderate MR)	0.12	(0.04–0.32)	<0.001**
≥ Moderate tricuspid regurgitation	0.47	(0.25–0.91)	0.024*

Table S3. STROBE Checklist

STrengthening the Reporting of OBservational studies in Epidemiology — Cohort Study

Manuscript: Mitral Regurgitation after TAVR: Sex Differences and Low-Flow Low-Gradient Aortic Stenosis

Item	STROBE Recommendation	Manuscript Response (Warner et al.)
	TITLE AND ABSTRACT	
**1a**	Indicate the study design with a commonly used term in the title or abstract	Methods states "single-center, retrospective, observational analysis"; abstract describes "single-center, observational study"
**1b**	Provide an informative and balanced summary of what was done and what was found	Abstract includes Background, Methods, Results, and Conclusions with primary findings
	INTRODUCTION	
**2**	Explain scientific background and rationale for the investigation	Introduction describes MR prevalence in TAVR, existing evidence gaps, and rationale for examining sex differences and LFLG AS
**3**	State specific objectives, including prespecified hypotheses	Final Introduction paragraph states aims: evaluate MR improvement and predictors in classic vs LFLG AS, with focus on sex differences
	METHODS	
**4**	Present key elements of study design early in the paper	First Methods sentence: "single-center, retrospective, observational analysis from the Cleveland Clinic TAVR database"
**5**	Describe the setting, locations, and relevant dates including periods of recruitment, exposure, follow-up, and data collection	Single center (Cleveland Clinic); January 2016 to December 2020; follow-up from procedure to last contact or death
**6a**	Give eligibility criteria, and the sources and methods of selection of participants; describe methods of follow-up	Inclusion: severe AS (AVA <1 cm2), moderate or greater pre-TAVR MR. Exclusions: valve-in-valve, non-AS indication, aborted procedures, missing key data
**6b**	For matched studies, give matching criteria and number of exposed and unexposed	N/A — no matching performed
**7**	Clearly define all outcomes, exposures, predictors, potential confounders, and effect modifiers; give diagnostic criteria	Primary endpoint: MR improvement to mild or less at first post-TAVR echo. MR graded per ASE integrative guidelines.

		LFLG defined: AVA <1 cm2, gradient <40 mmHg, SVI <35 mL/m2
**8**	For each variable, give sources of data and details of methods of assessment	Echo data from certified sonographers interpreted by board-certified echocardiographers. Clinical variables and mortality from Epic EHR
**9**	Describe any efforts to address potential sources of bias	Acknowledged in Limitations: retrospective design, no blinded MR review, surgical referral bias, missing MR mechanism/etiology
**10**	Explain how the study size was arrived at	Consecutive eligible patients 2016-2020 (N=344). No a priori power calculation; sample size determined by registry data. Acknowledged as limitation
**11**	Explain how quantitative variables were handled; describe groupings chosen and why	Continuous variables as mean ± SD. Binary logistic endpoint (MR improved to mild or less). LFLG defined by established hemodynamic thresholds
**12a**	Describe all statistical methods, including those used to control for confounding	Multivariable logistic regression with prespecified covariates; VIF >5 used to exclude collinear variables; Cox proportional hazards for survival; IBM SPSS v29
**12b**	Describe any methods used to examine subgroups and interactions	Prespecified LFLG subgroup analysis. Multiplicative interaction terms tested between EF, MR severity, mean gradient, and LAVi via likelihood ratio testing (all NS)
**12c**	Explain how missing data were addressed	Listwise deletion. Patients with missing key data excluded at enrollment. N=306 complete cases for logistic model. No imputation
**12d**	Describe how loss to follow-up was addressed	Follow-up defined as time from TAVR to last contact or death. Patients without post-TAVR echo excluded from survival analysis (N=313 included in KM analysis)
**12e**	Describe any sensitivity analyses	No formal sensitivity analyses prespecified. C-statistic (0.81, 95% CI 0.78-0.87, bootstrap 1,000 resamples) reported as post-hoc discrimination metric
	RESULTS	
**13a**	Report numbers of individuals at each stage of study	2,630 total TAVR procedures; 344 with moderate or greater MR included; 313 with post-TAVR echo included in survival analysis

**13b**	Give reasons for non-participation at each stage	Exclusion categories described in text. Specific numbers by category not tabulated
**13c**	Consider use of a flow diagram	No flow diagram included; enrollment described in text
**14a**	Give characteristics of study participants and information on exposures and potential confounders	Table 1: full baseline demographics, comorbidities, and echocardiographic characteristics stratified by classic vs LFLG AS
**14b**	Indicate number of participants with missing data for each variable of interest	Not separately tabulated; missing data handled by exclusion at enrollment; complete-case N reported in methods
**14c**	Summarize follow-up time	Follow-up through 3 years. KM curves at 0-3 years with number-at-risk tables. Mean follow-up not explicitly stated
**15**	Report numbers of outcome events or summary measures over time	MR improvement to mild or less: 182/344 (52.9%). Deaths: 103 overall, 56 LFLG, 47 classic AS. KM curves Figures 3-5 with number-at-risk
**16a**	Give unadjusted and confounder-adjusted estimates with precision (95% CI)	Tables 2-6: OR/HR with 95% CI from multivariable models. Tables S1-S2: univariate ORs
**16b**	Report category boundaries when continuous variables were categorized	MR severity graded none/mild/moderate/severe per ASE guidelines. LFLG thresholds defined (AVA <1 cm2, gradient <40 mmHg, SVI <35 mL/m2)
**16c**	Consider translating relative risk into absolute risk for a meaningful time period	Survival probabilities shown on KM curves at 3 years. Absolute event rates not separately tabulated
**17**	Report other analyses (subgroups, interactions, sensitivity)	LFLG subgroup analyses (Tables 3, 5). Multiplicative interaction terms tested and NS. C-statistic with bootstrap 95% CI reported
	DISCUSSION	
**18**	Summarize key results with reference to study objectives	Discussion opens with summary of MR improvement rates, sex disparities, LFLG AS differences, and mortality associations
**19**	Discuss limitations, including sources of potential bias or imprecision	Dedicated Limitations section: retrospective design, surgical referral bias, absent MR mechanism data, no blinded echo review, missing anatomic data, no mitral intervention tracking, OR 0.06 threshold effect, EPV 9.6 in LFLG subgroup

**20**	Give a cautious overall interpretation considering objectives, limitations, and multiplicity of analyses	Discussion contextualizes findings vs prior literature. Residual MR noted as potential marker rather than driver of mortality. Exploratory nature of interactions acknowledged
**21**	Discuss the generalizability (external validity) of the study results	Single-center design acknowledged. Tertiary referral population (2016-2020) described. External validation of logistic model not performed
	OTHER INFORMATION	
**22**	Give the source of funding and role of the funders	Funding: None. Author disclosures listed on title page (GW Reed: consulting honoraria from Abbott, Boston Scientific, Edwards Lifesciences)

N/A = not applicable to this retrospective observational cohort study design. Completed per STROBE guidelines for cohort studies (von Elm et al., Lancet 2007; Epidemiology 2007).
